# Improving the Detection and Management of Kidney Health in Primary Care

**DOI:** 10.1177/23743735241256464

**Published:** 2024-06-13

**Authors:** Breonny Robson, Gary Deed, Richard KS Phoon

**Affiliations:** 1 200102 Kidney Health Australia, Melbourne, Victoria, Australia; 2 Healthcare Plus Medical Centre, Coorparoo, Queensland, Australia; 3 2541 Monash University, Melbourne, Victoria, Australia; 4 Department of Renal Medicine, 198347 Centre for Transplant and Renal Research, Westmead Hospital, Westmead, Australia; 5 522555 Faculty of Medicine and Health, The University of Sydney, Sydney, Australia

**Keywords:** chronic kidney disease, albuminuria, awareness, patient-orientated, First Nations Australians

## Abstract

Chronic kidney disease (CKD) is a major cause of morbidity and mortality, contributing to approximately 20 000 deaths in 2021 in Australia. Importantly, progression of CKD can be substantially reduced if it is detected and treated early. Here we present the perspectives of a general practitioner (primary care physician), a nephrologist and a patient advocate on how the diagnosis and management of CKD in primary care could be improved. Early detection and treatment of CKD are impeded by limited patient awareness and knowledge, communication challenges between patients and doctors, and psychosocial issues, with these factors also interacting with, and exacerbating, each other. We make the following recommendations to help improve outcomes in patients with CKD: (1) identifying people at increased risk of CKD and ensuring they have a complete kidney health check (including estimated glomerular filtration rate, urine albumin-creatinine ratio and a blood pressure check) every 1−2 years; (2) using simple, nonconfrontational language and supportive resources to communicate with patients about kidney health; (3) implementing early treatment to slow the progression of CKD and avoid adverse cardiovascular disease outcomes; and (4) asking patient-orientated questions to support shared decision-making and empower patients to be active partners in their healthcare. We acknowledge that limited time is a major barrier to implementing these recommendations in primary care. Utilizing the expertise of the whole practice team, and adopting supportive technology to introduce efficiencies, are likely to be of benefit. By adopting these recommendations, we believe general practitioners have the opportunity to drive improved outcomes and quality of life for people living with CKD in Australia.

## Introduction to the Issue

Chronic kidney disease (CKD) is a major cause of morbidity and mortality in Australia, contributing to approximately 20 000 deaths in 2021.^1^ Three in 4 Australians are at increased risk of CKD and the disease is twice as common in First Nations Australians.^1,2^ CKD progression can be substantially reduced if it is detected and treated early.^2,3^ It is estimated that targeted early detection of CKD over the next 20 years could generate 164 956 years of healthy life, by preventing 38 200 premature deaths and 237 324 hospitalizations associated with cardiovascular disease (CVD).^4^ In Australia, key areas that may impede early detection and treatment of CKD include a lack of awareness and knowledge, challenges with communication or interactions between patients and primary healthcare providers due to poor health literacy and cultural/linguistic barriers, a lack of dedicated funding for targeted screening, and psychosocial issues.^5^ This article synthesizes the viewpoints of a general practitioner (GP), a nephrologist, and a patient advocate on areas in which the management of kidney health in Australian primary care could be improved.

## Key Factors for Consideration

Awareness of CKD and the factors that increase CKD risk are inconsistent. GPs generally have a good awareness that hypertension, diabetes, family history, obesity and smoking increase CKD risk.^6^ However, up to 90% of kidney function is lost before people experience symptoms, and patients’ awareness of their kidney health and the factors that increase CKD risk is relatively low.^2^ According to an Australian Health Survey, only 6.1% of patients with biomedical signs of CKD self-reported their condition.^7^ In a survey of patients with CKD (stages 1-5), at least 70% of patients identified hypertension, diabetes, and obesity as CKD risk factors; however, only 54% and 38% recognized that CKD was more common in people who smoked or were First Nation Australian, respectively.^6^ Furthermore, the patients’ awareness of comorbidities associated with CKD was lower than for other chronic conditions, and CKD was perceived to pose less of a threat to life than diabetes or CVD.^6^ Missed opportunities for targeted detection of CKD in high-risk groups is common, particularly in First Nations Australians, and can contribute to the late diagnosis and poor outcomes often seen in CKD.^8^

When it comes to treatment for CKD, people living with the condition are often not aware that medications they have been prescribed to treat hypertension, cholesterol, or diabetes also have kidney benefits.^5,6^ Furthermore, some patients may be concerned about the cost of medications and adverse effects, especially if they are taking multiple medications.^5^ This lack of understanding and awareness of CKD and its treatment can lead to feelings of disbelief regarding diagnosis, anxiety about disease progression, and uncertainty about prognosis.^5^ These feelings may be intensified if there is poor communication or interaction between the GP and patient.

## Recommendations

**Figure 1.**
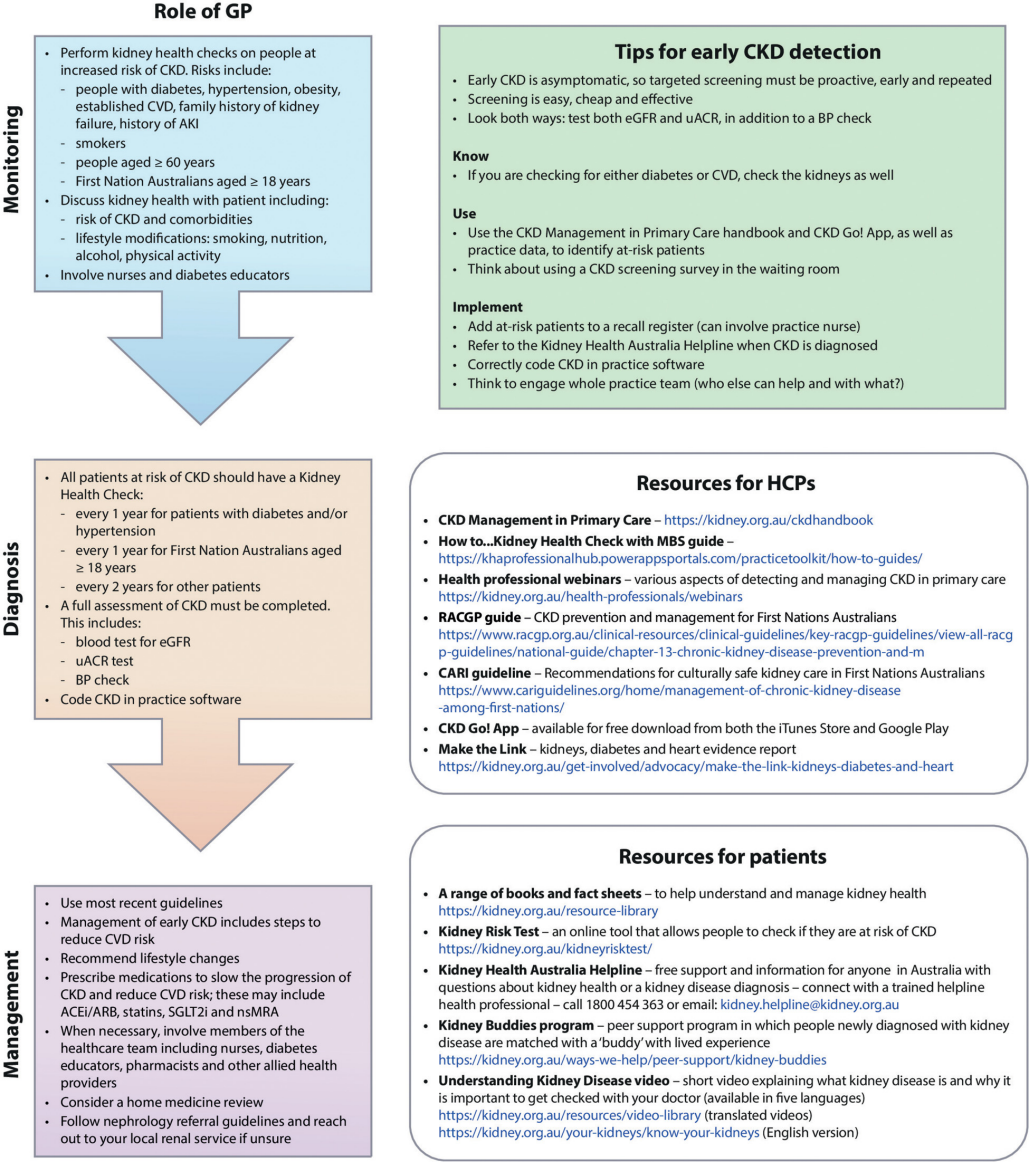
Tips for improving the detection and management of CKD in primary care. ACEi, angiotensin-converting enzyme inhibitor; AKI, acute kidney injury; BP, blood pressure; CKD, chronic kidney disease; CVD, cardiovascular disease; eGFR, estimated glomerular filtration rate; GP, general practitioner; HCP, healthcare professional; nsMRA, nonsteroidal mineralocorticoid receptor antagonist; SGLT2i, sodium-glucose cotransporter-2 inhibitor; uACR, urine albumin-creatinine ratio.

In recognition of these challenges, numerous resources have been developed to support and help educate GPs and patients in Australia on the importance of kidney health, the links between diabetes, CVD, and CKD (providing key context for treatment advances), and best practices for monitoring, diagnosing and treating CKD (Figure 1). Here, we provide key recommendations to assist GPs in communicating with patients effectively and to support early detection of CKD and successful disease management.

The kidney-conscious GP is educated in kidney health and understands their role in assessing CKD risk. It is important to conduct a full Kidney Health Check for all patients who are at increased risk of CKD every 1–2 years.^2^ This includes a blood test to assess estimated glomerular filtration rate (eGFR), a urine albumin-creatinine ratio (uACR) to check for albuminuria, and a blood pressure check (Figure 1).^2^ Unfortunately, the uACR is often missed in Australian general practice, which can lead to missed diagnoses and underestimation of CKD risk. uACR is a key component of targeted detection of kidney disease as it is essential for early diagnosis and staging of CKD, managing the overall risk of disease progression, and appropriate use of certain medications.

It is often challenging for GPs to discuss kidney health or a CKD diagnosis with their patients without alarming them (and potentially losing them from care). To find the right balance, the use of simple, nonconfrontational language, together with a collaborative, solution-focused approach between healthcare team and patient, may prove a useful tactic. For example, discussing a patient's “kidney problem” is less alarming than describing a patient's “weak or failing kidneys,”^9^ and “kidney health” may be more useful when discussing risk factors and lifestyle, rather than “kidney disease.” GPs may also consider referring to the percentage of kidney function (eg “kidneys are working at 50%”) rather than the stage of CKD, as the latter may alarm patients due to perceived similarity with stages of cancer.^10^

For all people with a diagnosis of CKD, management strategies aim to slow the decline of eGFR, reduce albuminuria, maintain blood pressure below 130/80, lower CVD risk and avoid further damage to the kidneys.^2^ Lifestyle modifications (stopping smoking, following a balanced nutritional plan, limiting alcohol intake and increasing physical activity) should be considered the first-line strategy, with the addition of medications to slow disease progression, reduce albuminuria, and manage blood pressure if indicated (Figure 1). Following a CKD diagnosis, correctly coding CKD in practice software will assist with medication considerations and can help improve the overall management of CKD leading to positive outcomes for patients. Effective management can help patients to feel better, delay disease progression and have a positive effect on CKD and CVD outcomes.^2^ This is best achieved by involving patients in the development of a personalized management plan.

GPs can consider asking patient-orientated questions to engage the patient in their management plan. For example, “Can we set goals for managing this together?” and “Do you think there are any challenges you might face in sticking with your management plan?”. Empowering patients and encouraging them to be part of the decision-making process, especially around lifestyle, will more likely result in lasting positive behavioral changes. GPs can also guide patients to patient-tailored resources that inform them about the rationale for their care. Patient-oriented questions may need to be adapted once the GP has a better understanding of the patient's personal barriers, beliefs, and preferences. It should also be acknowledged that the predominant health concerns and priorities, and perceived determinants of health, may vary between communities, and that GPs’ attempts to engage patients may benefit from an awareness of such differences.

With the many competing priorities in primary care, it is not uncommon for CKD to be de-prioritized by time-poor GPs who have a myriad of concerns to address in a short timeframe. Lack of time has long been a key factor limiting the effective implementation of guidelines by GPs,^11^ and has been identified as the biggest barrier to the detection and management of CKD in primary care.^12^ Utilizing the expertise of the whole practice team may be of benefit, as well as implementing practice systems to foster proactive detection and management of CKD. For example, primary healthcare nurses and diabetes educators may provide support in the identification of high-risk individuals, detection of CKD, and communication around diagnosis and treatment. Consistent with this, the presence of a collaborative relationship between members of the healthcare team has been identified as one of the most effective enablers of CKD detection and management,^12^ as well as helping to reduce clinical inertia.^13^ The presence of supportive technology is also a key enabler,^12^ and recent advancements in artificial intelligence may further enhance opportunities to utilize technology to achieve efficiencies in primary care. Examples include reducing the burden of routine administration and clinical tasks, improving diagnosis, and increasing patient engagement and compliance.^14–16^

## Conclusion

In summary, early diagnosis and management of CKD in primary care are critical in reducing disease progression. By implementing the key recommendations described here within their practice, we believe GPs have the opportunity to drive improved outcomes and quality of life for people living with CKD in Australia.
